# ASO Author Reflections: Failure to Cure in Patients Undergoing Surgery for Gastric Cancer: A Nationwide Cohort Study

**DOI:** 10.1245/s10434-020-09539-7

**Published:** 2021-01-27

**Authors:** Daan M. Voeten, Mark I. van Berge Henegouwen

**Affiliations:** 1 Department of Surgery, Amsterdam UMC, Cancer Center Amsterdam, University of Amsterdam, Amsterdam, the Netherlands; 2 Scientific Bureau, Dutch Institute for Clinical Auditing, Leiden, the Netherlands

## Past

Gastrectomy is the cornerstone of curative treatment for gastric cancer patients. It is an invasive procedure with considerable postoperative morbidity and mortality rates. Therefore, literature focuses on improving the quality of the surgical procedure. In 1992, Clavien et al. introduced a now broadly accepted complication severity score enabling greater uniformity in reporting surgical complications.^1^ Additionally, an outcome measure entitled “failure to cure” was introduced, defined as surgery not meeting its initial aim. However, until recently, literature describing failure to cure was lacking. A recent study described failure to cure as an important outcome measure in esophageal cancer surgery that was able to identify hospital variation and areas for improvement.^2^ Failure to cure not only reports on the quality of surgery but also reflects the quality of preoperative diagnostics, the selection of surgical and neoadjuvant therapy candidates, and evaluates the multidisciplinary team’s decision-making. For gastric cancer, outcome measures evaluating the quality of the entire process of care are not yet available.

## Present

The current population-based cohort study evaluated failure to cure as a composite outcome measure in gastric cancer surgery.^3^ It defined failure to cure as the following: (1) futile surgery due to intraoperative distant metastasis or local tumor irresectability, (2) microscopically or macroscopically incomplete resection (R1/R2), or (3) 30-day and/or in-hospital mortality. This study included patients from the Dutch Upper Gastrointestinal Cancer Audit (DUCA), a mandatory surgical audit including all gastric cancer patients undergoing surgery with the intent of resection in the Netherlands since 2011. Failure to cure occurred in 22.3% of 3862 gastric cancer patients undergoing potentially curative surgery. This rate ranged from 15 to 35% among Dutch hospitals, which indicates that nationwide improvement is possible and necessary. The composite outcome measure has statistical advantages in identifying outlier hospitals. It should, however, be used in addition to individual outcome parameters as these show clinicians’ actual areas for improvement. In addition, when using failure to cure, it is vital not to set the benchmark at 0% as this could potentially lead to risk-averse behavior. On the other hand, high failure to cure rates suggest that too many patients are exposed to unnecessary surgical risks.

In the Netherlands, there is increasing awareness of hospital variation in the administration of neoadjuvant therapy. The current study also showed that failure to cure rates are high in hospitals where neoadjuvant therapy is omitted relatively frequently. This strengthens the recommendation of neoadjuvant chemotherapy in the Dutch guideline. One could even argue that administration of neoadjuvant therapy is a proxy for the overall quality of multimodal care provided by a hospital.

## Future

Diagnostic laparoscopy potentially has a positive effect on preventing futile surgery and therewith reducing failure to cure. However, literature on the value of staging laparoscopy is not unambiguous, and a recent study showed that futile surgery rates after diagnostic laparoscopy were still as high as 16%.^4^ Outcomes of the prospective PLASTIC study, investigating the value of staging laparoscopy and fluorodeoxyglucose (FDG)-positron emission tomography (PET)/computed tomography (CT) in gastric cancer patients, are awaited.^5^ Its routine performance might reduce failure to cure rates.

Reduction of hospital variation in failure to cure might induce nationwide improvement of gastric cancer care. Greater uniformity in selecting patients eligible for systemic preoperative therapy or potentially curative surgery might be an important step towards realizing this improvement. This could be realized either through centralization or through enhancing uniformity by regional multidisciplinary team meetings in which multiple upper gastrointestinal specialists from multiple specialties and hospitals participate. Dutch upper gastrointestinal cancer surgeons organize yearly best-practice meetings and discuss hospital differences in outcomes, workup, and clinical practices. In this way, uniformity is enhanced, and hospital variation reduced at a national level.
